# Analysis of public responses to preparedness policies: the cases of H1N1 influenza vaccination and gas mask distribution

**DOI:** 10.1186/2045-4015-2-11

**Published:** 2013-03-27

**Authors:** Baruch Velan, Valentina Boyko, Gilead Shenhar, Liat Lerner-Geva, Giora Kaplan

**Affiliations:** 1 Genetic Policy and Bioethics Unit, Gertner Institute for Epidemiology and Health Policy Research, Tel-Hashomer 52621, Israel; 2 Women and Children’s Health Research Unit, Gertner Institute for Epidemiology and Health Policy Research, Tel-Hashomer, Ramat Gan, Israel; 3 Trauma & Emergency Medicine Research Center, Gertner Institute for Epidemiology and Health Policy Research, Tel-Hashomer, Ramat Gan, Israel; 4 Psychosocial Aspects of Health, Gertner Institute for Epidemiology and Health Policy Research, Tel-Hashomer, Ramat Gan, Israel; 5 Sackler Faculty of Medicine, Tel Aviv University, Ramat Aviv, Tel Aviv, Israel

**Keywords:** H1N1-influenza, Acceptance, Public-trust, Gas-Masks, Preparedness

## Abstract

**Background:**

During several months in 2009–2010, the Israeli population was asked to take part in two preparedness programs: Acquisition of gas masks against a potential chemical-warfare attack, and vaccination against the A/H1N1 influenza pandemics. Compliance with the first request was moderate and did not attract much attention, whereas compliance with the second request was very low and was accompanied by significant controversy. The aims of this study are to compare the public’s attitudes towards these two preparedness campaigns, and to explore the roles of trust, reasoned assessment, and reflexive reactions in the public’s response to governmental preparedness policies.

**Methods:**

The comparative analysis was based on a telephone survey of 2,018 respondents representing a cross-section of the adult Israeli population. Univariate analysis to describe associations of public response and attitude was performed by Chi-square tests.

**Findings:**

A set of queries related to actual compliance, trust in credibility of authorities, personal opinions, reasons for non-compliance, and attitudes towards uncertainties was used to characterize the response to mask-acquisition and vaccination. In the case of mask-acquisition, the dominant response profile was of trusting compliance based on non-conditional belief in the need to adhere to the recommendation (35.6% of respondents). In the case of vaccination, the dominant response profile was of trusting non-compliance based on a reflective belief in the need for adherence (34.8% of respondents). Among the variables examined in the study, passivity was found to be the major reason for non-compliance with mask-acquisition, whereas reasoned assessment of risk played a major role in non-compliance with vaccination. Realization of the complexity in dealing with uncertainty related to developing epidemics and to newly-developed vaccines was identified in the public’s response to the H1N1 vaccination campaign.

**Conclusions:**

The newly identified profile of “trusting-reflective-non-complier” individuals should be of concern to policy makers. The public is not accepting governmental recommendations in an unconditional manner. This is not driven by lack of trust in authorities, but rather by the perception of the responsibility of individuals in confronting forthcoming risks. Nevertheless, under certain conditions the public may respond in a non-reflective way and delegate this responsibly to authorities in an uncontested manner. This leaves the policy makers with the complex challenge of interacting with a passive non-involved public or alternatively with an opinionated, reflexive public.

## Background

The responsibility of the State for the health of its population entails appropriate preparedness against emerging large-scale health hazards. This includes risk communication as well as provision of the appropriate protection measures. During a period of several months during 2009–2010, the State of Israel exercised this responsibility by launching two national campaigns. The Israeli population was urged to get vaccinated against the developing H1N1 pandemic influenza, and to acquire gas masks and hoods against a potential future attack by chemical or biological warfare.

The A/H1N1 influenza (hereafter, H1N1) of 2009 is considered one of the most widespread pandemics in recent history. In March 2009, the outburst of a novel strain of influenza, linked to swine influenza, was detected in Mexico, and as of January 2010 it had caused nearly 15,000 deaths in 209 countries [1,2]. H1N1 was declared a pandemic by the World Health Organization on June 2009, as soon as infection had shown sustained human-to-human transmission in different geographic regions [3]. The WHO declaration boosted the implementation of various countermeasure programs by national organizations worldwide, and prompted the development and production of vaccines against the H1N1 virus [4]. The first H1N1 vaccines were licensed in mid-September, and by October 2009 most industrialized countries had rolled out national vaccination programs.

The sequence of the H1N1 events in Israel resembled those observed in other industrialized countries. The first case of H1N1 was identified in Israel on April 24, 2009, and by the end of July 2009, 1500 confirmed cases were reported. The Israeli government placed an order for 7.7 million doses at the earlier stages of the pandemic, and the vaccination program was launched at the beginning of November 2009. Vaccination was offered first to individuals at risk and health care workers, and at later stages to everyone, free of charge, supplying the vaccine progressively [5,6]. Vaccination was carried on by the Israeli HMOs, and was promoted extensively by the Ministry of Health. Controversies related to vaccination, focusing mainly on the safety of the adjuvant-containing inactivated vaccine preparations purchased by Israel started early on. The government’s major efforts to promote the H1N1 vaccine through a widespread campaign involving national TV, newspapers, leaflets, and posters placed in public areas were met with skepticism, resulting in a low compliance rates among Israelis [7].

The decision to provide gas masks to the entire Israeli population can be traced back to the period of the First Gulf War in 1991 [8]. Gas mask distribution was based on accumulating intelligence reports on Iraq’s chemical and biological warfare arsenals and on the actual fact that during the First Gulf War, Israel was attacked by 39 Iraqi missiles, all of which turned out to carry conventional missile heads.

The threat of a non-conventional attack reemerged in 2003 during preparations for the Second Gulf War. The Israeli Civil Population was called to acquire new and renewed masks. At a certain point during the war, the public was asked to prepare the masks for use by opening the mask boxes, connecting the filter, and adjusting the mask to the face. Israel was not attacked during the Second Gulf War and the masks were not used. Nevertheless, opening the packaged masks and filters led to their expiration and the need for renewal [9].

During 2007–2008 the masks were collected from the public for aftercare and in 2010 the Israeli government decided to distribute the re-validated gas masks to the Israeli population [10]. Distribution was commissioned to the Israeli Postal Company, which is a commercial enterprise. The distribution, which started in April 2010, was preceded by a two-week long national campaign on the three main TV channels. The campaign urged the population to re-acquire gas masks, offering two options for acquisition: by courier (with a modest payment of $6 per family), or by collecting the masks at appointed distribution points (free of charge).

The responses of the public to the H1N1 vaccination campaign and to the mask distribution campaign were quite different. The vaccination campaign met with much controversy, leading to a very intense public debate and to a very low compliance rate (about 13%) [7]. In contrast, the gas mask acquisition campaign did not lead to debates or discussions, and the compliance rate was moderate (approximately one-third).

The aim of the present study is to analyze the public’s attitude to H1N1 vaccination and gas mask acquisition in a comparative way. We realize that these two cases differ along several dimensions, including the severity of the threat and whether it is local or global, the risks and costs involved in adopting the preventive measure, and the nature of the opposition to adoption of the preventive measure. Still, it is useful and important to consider the two cases in a comparative context as they both relate to large-scale public health risks, and provide an opportunity to explore the complexity of the attitudes of the public toward governmental authorities in the face of such risks. This allows the identification of two modes of response to preparedness policies, one characterized by non-reflective compliance and the other by conscious noncompliance.

The study does not purport to be an exhaustive analysis of all the reasons for the incomplete adherence of the Israeli public to governmental recommendations regarding H1N1 and gas mask acquisition, nor does it seek to quantify the independent impact of each of the many factors involved. Instead, it seeks to identify and highlight several key factors that have previously not been noted in the international literature on public response to governmental preparedness policies.

## Methods

### Study population

This cross-sectional study was based on a randomly selected representative sample of the Israeli adult population (aged 21 and over). A probabilistic stratified sampling of households was built, based on official statistical areas characterized by socio-demographic characteristics. Areas were then matched with the computerized list of subscribers to the national telephone company, and households were randomly chosen. Excluded were fax numbers, disconnected numbers, and commercial numbers. As of 2009, about 85% of Israeli households had landline telephones [11].

### Data collection

The survey was conducted by telephone during March 2011 by the Cohen Institute for Public Opinion Research at the Tel Aviv University. The telephone interviews were conducted in Hebrew (79.5%), Arabic (15.7%), and Russian (4.8%).

The number of households in the target sample was 4,370; of these 1,112 contacts turned out to be abortive (fax numbers, disconnected numbers, failure to establish contact following up to five tries). Contact was established with 3,258 households. Of these, 1,240 did not agree to take part in the survey. The final sample included 2,018 complete questionnaires, yielding a response rate of 62%. Only one adult in each household was interviewed. This group was also used in a different study addressing responses to other recommendations, including Human Papilloma Virus vaccination, childhood vaccination, and travel vaccines (not presented here).

All interviewees were asked to respond to a series of questions relating to their attitudes towards preparedness against the H1N1 influenza epidemic. In parallel, interviewees were asked about their attitude toward preparedness against a potential non-conventional attack by chemical warfare, and attitudes towards governmental decisions. All participants were also asked about their actual compliance with flu vaccination and gas mask acquisition, and asked to provide explanations for non-compliance with recommendations. The design of the study as well as the questionnaire used was approved by the ethics committee of the Sheba Medical Center.

### Statistical analysis

Statistical analysis was performed using SAS statistical software (Version 9.2, SAS Institute Inc., Cary, NC). Categorical variables were compared by chi-square tests. A two-sided *p* value <0.05 was considered significant. Degree of agreement between attitudes to different recommendations was measured by calculating Kappa coefficients.

## Results

### Survey sample

The survey sample included 2,018 adult participants. The socio-demographic characterization of the survey group included: gender (46% males, 54% females), income (38% below average, 23% average, 26% above average, 13% non-disclosure), ethnicity (18% Arabs; 82% Jews), education (43% with ≤ 12 years of schooling; 57% with > 12 years of schooling), and age (34% aged 21–39, 36% aged 40–59, 30% aged 60 and older). Representation of the main socio-demographic variables in the sample was not significantly different from their representation in the adult Israeli population [12] in terms of gender, income, and ethnicity. The exceptions were the over-representation of the ≥60 age group (30% vs. 22% in the general population), and the over-representation of individuals with ≥12 years of schooling (57% vs. 47% in the general population).

### Rates of Compliance with H1N1 vaccination and mask acquisition

The survey was conducted during March 2011. All 2,018 participants in the survey were asked if they had been vaccinated against H1N1 influenza during the winter of 2009, and if they had acquired a gas mask during the 12 months preceding the survey. About 22% of the respondents declared that they had complied with the recommendation for H1N1 vaccination, whereas 56% declared that they had complied with the recommendation for gas mask acquirement (Table 1). These rates are higher than the actual compliance rates of the Israeli population, which at that point in time were 13% for H1N1 vaccination [Communicated by Prof. I. Grotto, head of the Public Health Services of the Israeli Ministry of Health] and about 32% for gas mask acquisition when the survey was conducted [Communicated by the spokesman of the Israeli Defense Forces]. It is interesting to note that for both recommendations the same divergence was noted: ~1.7-fold ratio between actual compliance of the Israeli population and declared compliance of the survey group. This bias could be related to social desirability (responders’ attempts to provide a response that will presumably please the interviewer), or to a compliance bias in the selection of the survey group (individuals who comply with health recommendations are also the ones that will most likely agree to take part in surveys).

**Table 1:** Rates of compliance with H1N1 vaccination and gas mask acquisition among 2018 responders

**Actual compliance**	**H1N1 vaccination**	**Gas mask acquisition**
	n **(%)**	n **(%)**
Complied	442 **(21.9)**	1132 **(56.1)**
Did not comply	1555 **(77.1)**	836 **(41.4)**
No answer	21 **(1.0)**	50 **(2.5)**

### Public’s perceptions of authorities’ motivations in promoting vaccination and gas mask acquisition

The H1N1 vaccination campaign was launched by the Ministry of Health (MOH), whereas that for mask acquisition was launched by the Home Front Command (HFC). The notable difference in compliance to these two campaigns may be related, in part, to differences in the public’s attitudes towards these two institutions. To examine this, respondents were asked to express their opinion on the motivation of the authorities in launching the two campaigns. Four different answers were offered as response options (stated in Table 2).

**Table 2:** Public’s perceptions of the motivations of the authorities in prompting vaccination and gas mask acquisition (N = 2018)

**Attitude towards authorities**	**H1N1 vaccine**	**Gas masks**	**Perceived reasons for launching campaign**	**H1N1 vaccine**	**Gas masks**
	**%** n	**%**n		n	n
**Trust** – Serving public interests ^a^	**62.9%** 1269	**64.4%** 1299			
			1) Authorities forecasted outbreak/attack and wanted to protect us	615	457
			2) Authorities were uncertain yet wanted to protect us	654	842
**Distrust** – Serving other interests ^b^	**30.4%** 614	**25.7%** 519			
			3) Authorities were uncertain and wanted to be covered	407	356
			4) Authorities were influenced by ulterior interests	112	258
Not defined	**6.7%** 135	**9.9%** 200	No response	135	200

^a^ Defined as choosing answers 1 or 2.

^b^ Defined as choosing answers 3 or 4.

Two response options attribute to authorities a sincere intention to serve the public’s interest, and thus express trust and credibility. One of those responses (1) suggests that the authorities have forecasted a real upcoming danger and attempted to provide protection against it. The other response (2) suggests that authorities were uncertain about the developing danger, yet believed that countermeasures should be provided in order to protect the public. The majority of the responders believed that the authorities were motivated by the desire to serve the public in launching both the H1N1 vaccination campaign and the mask campaign (62.9%, 269 respondents; and 64.4%, 229 respondents, respectively). While the percentage of individuals believing in the credibility of authorities is practically identical for both recommendations (*p* = 0.16), uncertainty in the developing danger (response 2) is associated, to a greater extent, with the mask campaign than with the vaccination campaign (41.7%, 842; and 32.4%, 654, respectively; <0.0001).

The two other response options relate to actions that are not necessarily motivated by a desire to serve public interest. One of those response options (3) suggests that authorities were uncertain about the danger, but acted according to the worst case scenario in order to be protected from future criticism if the danger eventually materializes. The other response option (4) attributes ulterior motives to authorities. The choice of either of these two response options expresses distrust in authorities. Such distrust was expressed by 30.4% of the respondents (614) when addressing the vaccination campaign, compared to 25.7% (519) in the case of the mask campaign (*p* < 0.0001). The agreement in the attitudes of specific individuals to authorities’ motivations in promoting vaccination versus mask acquisition was found to be significant, yet moderate. The number of observed agreements was 1189 (58.9%), and the calculated kappa coefficient was 0.20 (95% CI = 0.16–0.23).

Taken together, these results attest to a rather high degree of trust in the credibility of authorities’ decisions related to both campaigns. This also suggests that the difference in compliance between H1N1 vaccination and gas mask acquisition cannot be attributed to major differences in the attitudes towards the MOH and the HFC. Indeed, analysis of the relationship between trust in authorities and actual compliance reveals that distrust is a poor predictor of non-compliance (Table 3). While the majority of compliers with vaccination and gas mask acquisition also expressed trust in authorities (78.7%, 348; and 70.7%, 800, respectively), non-compliance was not necessarily associated with distrust. As many as 58.6% of the responders (911) who did not comply with the H1N1 vaccination recommendation did so despite their belief in the credibility of the authorities. In a similar way, 57.9% (484) of the non-compliers with mask-acquisition appear to exhibit trust towards authorities.

**Table 3:** Interrelationships between trust in authorities and compliance with vaccination and gas mask acquisition (N = 2018)

	**H1N1 vaccine **^**a**^		**Gas masks **^**b**^	
**Compliance**	**Complied**	**Did not comply**	**Complied**	**Did not comply**
	N=442	N=1555	N=1132	N=836
**Attitude**	n **(%)**	n **(%)**	n **(%)**	n **(%)**
**Trust authorities**	348 **(78.7)**	911 **(58.6)**	800 **(70.7)**	484 **(57.9)**
**Distrust authorities**	75 **(17.0)**	535 **(34.4)**	249 **(22.0)**	264 **(31.6)**
**Not defined**	19 **(4.3)**	109 **(7.0)**	83 **(7.3)**	88 **(10.5)**

^a^ 21 participants not disclosing compliance were not included.

^b^ 50 participants not disclosing compliance were not included.

Additional insight into the interrelationship between trust and compliance can be gained by grouping the population into distinct subgroups: “trusting compliers”, “trusting non-compliers”, “distrusting compliers”, and “distrusting non-compliers”. In the case of vaccination, “trusting-non-compliers” form the largest group: 911 of the respondents who comprise 45.1% of the survey group (Table 3). These are followed by “distrusting-non-compliers” (26.5%, 535), “trusting compliers” (17.2%, 348), and “distrusting-compliers” (3.7%, 75). A similar analysis of the response to mask-acquisition reveals that the leading group is that of “trusting compliers”: 800 respondents who comprise 39.6% of the survey population, while only 484 respondents (24%) can be defined as “trusting-non-compliers”. These results suggest that trust can be translated into different modes of action, depending on the specific circumstances.

### Attitudes towards compliance with H1N1 vaccination and mask acquisition

The notable difference in the behavior of the public in response to the vaccination campaign and the mask campaign could be due, in part, to differences in the public’s attitudes towards these two procedures. To examine this, all respondents were asked to express their attitudes towards compliance with vaccinations or mask acquisition by choosing one of four response options: 1) everyone should comply; 2) people at risk should comply; 3) compliance should be left to personal choice; 4) there is no need to comply (Table 4). The first option could be considered as a tendency towards “Non-conditional Acceptance”, the second and third options could be considered as a tendency towards “Reflective Acceptance”, whereas the last choice expresses “Non-Acceptance”.

**Table 4:** Public’s attitude towards acceptance of H1N1 vaccination and mask acquisition (N = 2018)

**Favorable attitude**	**H1N1 vaccine (%)**	**Gas masks (%)**	**Response option**	**H1N1 vaccine** n	**Gas masks** n
**Non-conditional acceptance **^**a**^	**23.3**	**75.0**		470	1515
			1) Everyone should have complied		
**Reflective acceptance **^**b**^	**72.5**	**15.4**			
			2) People at risk should have complied	587	75
			3) Compliance is a matter of personal choice	877	234
**Non-acceptance **^**c**^	**2.2**	**4.6**			
			4) There was no need to comply	44	94
**No response**	**2.0**	**5.0**	No opinion	40	100

^a^ Defined as choosing response option 1.

^b^ Defined as choosing response option 2 or 3.

^c^ Defined as choosing response option 4.

The distribution of responses (Table 4) indicates that “Non-Acceptance” is a minor attitude-motive for both recommendations (2.2%, 44 for vaccination; and 4.6%, 94 for masks). The vast majority of respondents expressed acceptance of the two recommendations, yet the nature of the acceptance was very different. In the case of gas mask acquisition, 75% of the respondents expressed “Non-conditional Acceptance” and 15.4% expressed “Reflective Acceptance”. This ratio was reversed in the case of the attitudes towards H1N1 vaccination. Here, only 23.3% favored “Non-Conditional Acceptance” whereas 72.5% believed that acceptance should be linked to either the personal choice of the individual or the specific risk faced by each individual (“Reflective Acceptance”).

It appears that acceptance of H1N1 vaccination is a rather reflective process and is associated with the pre-existence of specific health conditions. In contrast, acquisition of gas masks is being viewed as a procedure that does not require contemplation and analysis. Interestingly, 43% of the respondents (877) believe that H1N1 vaccination should be a matter of personal decision, yet only 11% (234) believe that this is the case for gas mask acquisition.

The analysis of interrelationships between attitude and compliance (Table 5) reveals a varied interrelationship. In the case of mask-acquisition, most compliers (86.2%) believe in “Non-Conditional Acceptance”, but this is also the case for the majority of non-compliers (62.8%). In the case of vaccination, compliers are equally divided between those who favor “Non-conditional Acceptance” and “Reflective Acceptance” (48.2% and 48.9%). Notably, non-compliance to vaccination is associated with a belief in “Reflective Acceptance” (79.4%) rather than “Non-Conditional Acceptance” (16.3%).

**Table 5:** Interrelationships between favorable attitude and compliance with vaccination and mask acquisition (N = 2018)

	**H1N1 vaccine**^**a**^		**Gas masks**^**b**^	
**Compliance**	**Complied** N=442	**Did not-comply** N=1555	**Complied** N=1132	**Did not-comply** N=836
**Attitude**	n **(%)**	n **(%)**	n **(%)**	n **(%)**
**Non-conditional acceptance**	213 **(48.2)**	253 **(16.3)**	976**(86.2)**	525 **(62.8)**
**Reflective acceptance**	216 **(48.9)**	1235**(79.4)**	123 **(10.9)**	181 **(21.7)**
**Non-acceptance**	2 **(0.4)**	42 **(2.7)**	17 **(1.5)**	74 **(8.8)**
**No response**	11 **(2.5)**	25 **(1.6)**	16 **(1.4)**	56 **(6.7)**

^a^ 21 participants not disclosing compliance were not included.

^b^ 50 participants not disclosing compliance were not included.

Grouping of the respondents according to compliance versus attitude (Table 5), reveals that in the case of vaccination the dominant group can be defined as “reflective non compliers”, comprising 1235 of the 2018 respondents (61.2%), whereas for gas masks the dominant group consists of “non-conditional acceptors”, which consists of 976 respondents (48.4% of the survey group).

### Profiling of responses to recommendations for H1N1 vaccination and mask-acquisition

The intricate interrelationship among compliance, attitude, and trust, led us to profile the respondents based on variants related to these three markers. This was achieved by cross-tabulation of Tables 3 and 5 to generate new composite tables, defining response profiles to mask-acquisition (Table 6) and vaccination (Table 7). This allowed the grouping of the survey population into 20 subgroups; 12 groups were found to be small in proportion or ill-defined, leaving us with 8 groups.

**Table 6:** Profiling of public’s response to recommended gas mask acquisition (N = 2018)

**Compliance & trust**	**Trusting compliers** N=800	**Trusting non-compliers** N=484	**Distrusting compliers** N=249	**Distrusting non-compliers** N=264	**Undefined group **^**a**^ N=221
**Attitude**	n **(%)**	n **(%)**	n **(%)**	n **(%)**	n **(%)**
**Non-conditional acceptance**	719 **(90.0)**	348 **(71.9)**	193 **(77.5)**	133 **(50.4)**	122 **(55.2)**
**Reflective acceptance**	69 **(8.6)**	90 **(18.5)**	44 **(17.7)**	74 **(8.0)**	32 **(14.5)**
**Non-acceptance**	6 **(0. 7)**	23 **(4.7)**	9 **(3.6)**	42 **(15.9)**	14 **(6.3)**
**No response**	6 **(2.7)**	23 **(4.7)**	3 **(1.2)**	15 **(5.7)**	53 **(24.0)**

^a^ Did not respond to queries related to compliance or trust.

**Table 7:** Profiling of public’s response to recommended H1N1 vaccination (N = 2018)

**Compliance & trust**	**Trusting compliers** N=800	**Trusting non-compliers** N=484	**Distrusting compliers** N=249	**Distrusting non-compliers** N=264	**Undefined group **^**a**^ N=221
**Attitude**	n **(%)**	n **(%)**	n **(%)**	n **(%)**	n **(%)**
**Non-conditional acceptance**	177 **(50.9)**	190 **(20.9)**	190 **(20.9)**	27 **(36.0)**	24 **(16.1)**
**Reflective acceptance**	162 **(46.5)**	702 **(77.1)**	45 **(60.0)**	45 **(60.0)**	99 **(66.4)**
**Non-acceptance**	2 **(0. 6)**	10 **(1.0)**	0 **(0)**	24 **(4.5)**	8 **(5.4)**
**No response**	7 **(2.0)**	9 **(1.0)**	3 **(4.0)**	3 **(0.6)**	18 **(12.1)**

^a^ Did not respond to queries related to compliance or trust.

Analysis of the response to recommendations for mask acquisition (Table 6) reveals a predominant attitude of trust in authorities and a belief in non-conditional acceptance. In most cases this is translated into actual compliance, forming a large group of 719 “Trusting-non-conditional-compliers” (35.6% of the survey group). Nevertheless a smaller, yet substantial, group of respondents (348; 17.2%) who trust authorities and believe in non-conditional-acceptance chose not to comply. All other permutations on compliance/attitude/trust were observed as well (Table 6), yet these were represented by groups of small percentage (below 10%).

The response to recommendation for H1N1 vaccination (Table 7) is characterized by a different attitude profile. The dominant attitude here is trust in authorities and belief in conditional reflective acceptance. In most cases this is translated into non-compliance, forming a large group of 702 respondents defined as “Trusting-reflective-non-compliers” (34.8% of responders). A smaller group of non-compliers (456; 22.6%) distrust authorities and believe in reflective acceptance (“Distrusting-reflective-non-compliers”). Other groups were again represented in low proportions.

### Reasons for not complying with H1N1 influenza vaccination

To further analyze the reflective nature of the response towards H1N1 vaccination, the actual reasons for not complying with H1N1 influenza vaccination were examined. All 1555 respondents who did not receive the vaccine were asked to specify the reason for not doing so. Respondents were offered a set of 7 fixed responses to choose from. The fixed responses were based on the predominant answers provided previously [7] to an open-ended question about reasons for not complying with H1N1 vaccination. If none of the fixed responses expressed their motivation, respondents were allowed to phrase their own answer. Fixed answers as well as open answers were then grouped into three major categories: Passivity, Reasoning, and Distrust (Table 8).

**Table 8:** Reasons for not complying with H1N1 vaccination (N = 1555)

** Category**	n**(%)**	**Reason for non-compliance with flu vaccination**	**n**
**Passivity**	283 **(18.2)**	It just did not work out for me	225
		Open answers reflecting indifference	58
**Reasoning**	947 **(60.9)**	Decided to take the chance	89
		Did not feel in danger	426
		Think that interventions is not effective	133
		Intervention is harmful	213
		Open answers reflecting Reasoning	86
**Distrust**	257 **(16.5)**	I don’t believe in vaccines	187
		I did not trust authorities	57
		Open answers reflecting distrust	13
**Undefined**	68 **(4.4)**	Open answers of other category	44
		Reason not disclose	24

^a^ Please note that “belief” in Hebrew means “faith/conviction” rather than “opinion”.

Passivity was manifested by 283 respondents out of 1555 (18.2%). Reasoning was manifested by as many as 947 respondents out of 1555 (60.9%), and distrust by 257 respondents out of 1555 (16.5%). It should be noted that 68 (4.4%) participants failed to provide a defined reason for their action (Table 8). These observations suggest that, as indicated previously [7], the major motives in failure to get vaccinated can be traced to reasoned assessments of risks and benefits.

This behavior (Table 8), taken together with the prevalence of an attitude of “Conditional Acceptance” towards H1N1 vaccination (Table 4), suggests that the majority of the respondents did not object to vaccination, but felt that the evolvement of the H1N1 epidemic did not justify their personal compliance.

### Motives in the response to gas mask acquisition compared to H1N1 vaccination

All 836 respondents who did not acquire gas masks were asked to specify the reason for not doing so. Respondents were offered a set of five fixed responses to choose from and were also allowed not to disclose their reasons (Table 9). Indifference was manifested by as many as 50.8% (425) of the respondents, whereas distrust was manifested by only 4.7% (39). Reasoning was not a major motive in not complying with mask acquisition; as this applied to only 19.6% (169) of the respondents.

**Table 9:** Reasons for not complying with gas mask acquisition (N = 836)

**Category**	**n(%)**	**Reason for non-compliance with gas mask acquisition**	**n**
**Passivity**	425 **(50.8)**	It just did not work out for me	425
**Reasoning**	164 **(19.6)**	Decided to take the chance	27
		Did not feel in danger	72
		Think that Intervention is not effective	65
**Distrust**	39 **(4.7)**	I don’t trust the authorities	39
**Undefined**	208 **(24.9)**	Reason not disclosed	208

The response of the survey group to the recommendation related to gas mask acquisition was quite different from the response related to H1N1 vaccination (see summary in Table 10). The major motive in the response to gas mask acquisition was adherence with the recommendation (56%; 1123) and this was followed by non-adherence base on indifference (21%; 425). Non-adherence based on reasoning or on distrust appears to be of much lower incidence (8%, 164; and 2%, 39, respectively). In contrast, non-adherence based on reasoning appears to be the dominant motive (47%, 947) in the response to H1N1 vaccination, whereas non-adherence based on indifference appears to be of a lower prevalence (14%, 283), and resembles the prevalence of non-adherence based on distrust.

**Table 10:** Major motives in the actual response to recommendations related to H1N1 vaccination and gas mask acquisition (N = 2018)

**Major motive**	**Motives in acceptance of H1N1 vaccination**	**Motives in acquisition of gas masks**
	n **(%)**	n **(%)**
Adherence	442 **(21.9)**	1132 **(56.1)**
Non-adherence based on passivity	283 **(14.0)**	425 **(21.1)**
Non-adherence based on reasoning	947 **(46.9)**	164 **(8.1)**
Non-adherence based on distrust	257 **(12.8)**	39 **(1.92)**
Undefined non adherence	68 **(3.4)**	208 **(10.3)**
No response	21 **(1.0)**	50 **(2.50)**

### The recommendations of the public for preparedness measures against an emerging epidemic

The rational attitude exhibited by the respondents towards H1N1 vaccination together with the low compliance with recommendations suggest that the Israeli public believes in its ability to make the right decisions when dealing with epidemics. To further evaluate this, respondents were asked to provide their recommendations for national preparedness against an emerging outbreak. Two scenarios were offered to the respondents, both of which described a serious epidemic that was supposedly developing in a South American country. In one case the outbreak could be controlled by a well-established vaccine, and in the other case control could be achieved by using a newly developed vaccine. For both scenarios the respondents were asked to state their opinion on purchasing a national vaccine stockpile and on preventive vaccination. Four possible operational options were presented to the public (Table 11). One of the options represents a “wait and see” policy, suggesting that the state should wait and not purchase the vaccine. Another option is a “get prepared” option, suggesting that vaccines should be purchased but not used. The two other options represent an active approach (vaccinate the population); one suggests that individuals at risk should be vaccinated and the other that everyone should be vaccinated.

**Table 11:** Public’s opinion on preparedness policy to an epidemic emerging overseas (N = 2018)

**Recommended policy**	**Old vaccine (%)**	**New vaccine (%)**	**Answer**	**Old vaccine** n	**New vaccine** n
**Wait and see **^**a**^	**27.7**	**48.5**			
			1) State should monitor epidemic and not procure vaccine	559	978
**Get prepared **^**b**^	**22.9**	**15.5**	2) State should procure vaccine but not vaccinate yet	462	313
**Vaccinate **^**c**^	**39.8**	**27.9**			
			3) State should procure vaccine and vaccinate people at-risk	603	440
			4) State should procure vaccine and vaccinate everyone	200	123
Unknown	**9.6**	**8.1**	No opinion	194	164

^a^ Defined as choosing answer 1.

^b^ Defined as choosing answer 2.

^c^ Defined as choosing answers 3 or 4.

For both scenarios, the majority of respondents did not favor an active vaccination policy (about 40% favored vaccination in the case of an established vaccine, and less than 30% in the case of a new vaccine), and the number of respondents that favor vaccination of everyone was low (less than 10%). A large number of respondents favored a “wait and see” approach expressing a clear distinction between policies related to established vaccines and new vaccines. In the case of a new vaccine, 48.5% of the respondents (978) suggested that the state should refrain from purchasing the vaccine as opposed to 27.7% (559) in the case of an established vaccine (*p* < 0.0001).

The option of purchasing vaccine but not using it was favored by a substantial number of respondents. This option was more acceptable in the case of an established vaccine than in the case of a new vaccine (22.9%, 462; and 15.5%, 313, respectively; *p* < 0.0001).

Taken together, these observations suggest that the public is strongly opinionated about vaccination policy (note the low number of respondents who did not express an opinion on the subject, Table 10). Moreover, the public appears to appreciate the complexities and ambiguities related to epidemic preparedness policy.

## Discussion

Preparedness and response to potential large-scale health hazards constitutes one the biggest challenges for policy makers. This challenge is intensified as the certitude about the events and about the outcomes decreases. The accepted practice in many of such cases is to rely on the precautionary principle [13]. This principle establishes an obligation for action; even when the absence of scientific certainty makes it difficult to predict the likelihood of harm occurring, or the level of harm should it occur. In most cases this obligation has to be translated into “institutional” actions such as budget allocation, organization, and active preparedness of the relevant institutions. In certain cases the collaboration of the general public is required as exemplified by the two cases examined here: preparedness against pandemic flu and against a chemical warfare attack. This adds to the complexity of the preparedness challenge, since success is dependent on the compliance of the public.

The willingness of the public to take part in the preparedness efforts depends on the trust that the population has in authorities [14,15], on the way the public perceives the risk and the proposed countermeasures, and finally on the self-efficiency [16] of individuals, which determines their readiness to perform the active procedure of compliance. As a consequence, several modes of interrelationships between the public and the authorities can be expected.

On one hand, the public may exhibit distrust in authorities, reject expert recommendations altogether and as a consequence decide not to comply. Surprisingly, this was a rare phenomenon in the response to the two campaigns: 2.2% for mask-acquisition and 1.1% for H1N1 vaccination. On the other hand, the public may exhibit confidence in authorities, accept unconditionally the evaluations of formal experts, and exhibit high levels of compliance with governmental recommendations. This combination was manifested by a large number of individuals in their response to the mask acquisition campaign (35.6% of respondents) but was not common in the response to H1N1 vaccination (8.8%).

Our study reveals that the interrelationship between authorities and the public in the response to preparedness programs is more complex. In the case of mask-acquisition, a substantial fraction of the population (17.2%) believes in the credibility of the authorities, believes in the use of gas masks as a countermeasure, but nevertheless fail to actually comply with the recommendation. This discrepancy between beliefs and compliance with gas mask acquisition was found to be linked with low self-efficacy and passivity, an attitude often observed in the reaction to various health recommendations [17].

Contrary to this, low compliance with H1N1 vaccination is mainly related to a divergent evaluation of the situation, rather than passivity. Many respondents (34.8%) trusted the credibility of authorities, believed that one should accept vaccination under certain conditions, yet decide not to comply with vaccination.

Taken together, these observations suggest a major difference in the response towards the recommendation to acquire gas masks and that to get vaccinated against H1V1. The dominant response to the first recommendation can be defined as “trusting-non reflective-compliance”, whereas the dominant response to the second recommendation was “trusting-reflective-noncompliance”. This difference can be linked in the apparent difference in the attitude of the public to the two recommendations. The attitude towards H1N1 vaccination is characterized by reasoned assessment of the risks involved in failure to get vaccinated. Many people believe that not everyone needs to be vaccinated and that compliance should be left to personal decision. Moreover, when non-compliers were asked to explain their lack of compliance, about half of the respondents provided reasoned argumentation, based on their personal evaluation of risks related to disease and vaccine.

Contrary to this, the response to the gas mask campaign appears to lack sophistication. Only a small number of respondents believed that mask-acquisition is a matter of personal choice (perhaps in part because, as opposed to situation with vaccinations, mask use by one person has no bearing on other individuals). Surprisingly, respondents did not appreciate the relativity of risk (geographic distribution) as a factor in mask-acquisition. This is in contrast to the perception of risk-relativity, which was exhibited by the extensive self-evacuation of the population from Tel-Aviv to Jerusalem during the First Gulf War [18,19].

The marked difference in the response to H1N1 vaccination and gas mask acquisition can be explained in several ways. The difference can be attributed, in part, to the nature of the recommended intervention: In contrast to acquiring masks, which is merely a procedural event (getting to the distribution center or ordering the mask by phone), vaccination involves a non-pleasant physical intervention, entailing certain risks. Nevertheless, this difference in itself is not a sufficient explanation, since perception of real risk does not deter Israelis from accepting routine, well-established vaccination programs such as childhood vaccination [20,21]. Another explanation could be a differential attitude towards policies related directly to health and those elated to military defense [22]. Our results suggest, however, that the credibility of the MOH did not differ from the credibility of the HFC. It should be noted that the rejection of vaccination by the public, in general, is a well-recognized phenomenon that accompanies the history of vaccination and is prevalent in many different societies. The rejection of H1N1 vaccine, in particular, was identified in most industrialized countries and the controversy was propagated by popular media worldwide. Interestingly, the controversy over H1N1 vaccination was also manifested by the attitude of health care workers who often refused to get vaccinated. In contrast, the gas mask acquisition campaign was not associated with any notable controversy and was not criticized by the relevant experts.

One possible explanation for the difference in the attitudes towards H1N1 vaccines and gas-mask campaigns could be related to the time-dimensions framing the events. A potential chemical attack is remote and uncertain, and therefore individuals were not faced with the need to make an immediate decision or invest “intellectual efforts” in such a decision. On the other hand, the H1N1 epidemic was an evolving event [23,24], with new information accumulating every day, much uncertainty about future development, continuous debates among experts, and intense exposure by the media. This could lead to a high level of involvement of lay individuals and pressure to personally confront the vaccination dilemma. All this is reflected in the high awareness, high degree of reasoning, and a reflexive attitude towards the recommendation to comply with vaccination.

Several limitations can be identified in this study: (a) The study was based on a telephone survey and a time when 15% of the population did not have land-line phones; the response rate was 62%. (b) The divergence between the actual compliance of the Israeli population and declared compliance of the survey group is considerable, which could be attributed to social desirability or a sampling bias. This should be taken into consideration in the analysis of data. One should note, however, that this study suggests conformism in the public’s response towards mask-acquisition as opposed to non-conformism towards H1N1 vaccination. The potential biases described suggest that non-conformist approaches may actually be more prevalent among the full population than they were among our sample. Accordingly, this point actually strengthens the case for our contention that non-conformism plays a major role in the public response to governmental preparedness policies. (c) There are no analyses to determine if any of the responses to the survey questions differ by demographic variables. In our previous studies, we thoroughly examined the effect of demographic variants on attitudes towards vaccination [7,20], and demonstrated that non-conformism was associated with younger age and with the Jewish population. In this study, where compliance was juxtaposed to attitude and to trust, the large number of response variables (see Tables 6 and 7) would prohibit a coherent analysis of the effect of demography.

It should be stated that the attitude of the public to vaccination in general, and to H1N1 vaccination in particular has been the subject of many studies [22,23,25-29], using a variety of models and identifying a broad range of correlates of compliance. Those correlates include the extent to which H1N1 influenza is perceived to be risky, the extent to which the vaccination is perceived to be risky, and the degree of trust in government. We chose to analyze our data using a model that focuses on the role of reflexivity in the public’s response to risk [20,24,30,31]. Thus, the survey did not cover items related to compliance with other vaccines (seasonal influenza vaccines) or to exposure to information provided by formal authorities, nor did it examine in-depth the perception of risks related to vaccination or to diseases.

In the future, it would be useful to develop an integrated model of the public’s response to governmental preparedness policies that incorporates both the new factors identified in this article and the more widely recognized factors emphasized in the professional literature to date. It will also be useful to apply such an integrated model in empirical studies of public responses in various types of large-scale health hazards. This could be used to assess how the relative strengths of the possible explanatory factors vary across different types of hazards and societal contexts.

Notwithstanding all these limitations, the major contribution of this study is the identification of a unique interrelationship between trust in authorities, attitudes towards acceptance, and actual compliance with vaccination, which could be defined as a “trusting-reflective-noncompliance” response. The interrelationship between trust and compliance has been the subject of previous studies [25,26,32] and the interrelationship between a reflective attitude and compliance with vaccination has been analyzed in the past by us [7,20,24] and by others [27-29]. However, to the best of our knowledge, the analysis of the three-way interaction between trust, attitude, and compliance is new.

The newly identified “trusting-reflexive-noncompliance” response profile appears to be an outcome of the shift in responsibility from the State to the individual, which characterizes the era of the so called “reflexive modernity” [30,31]. In the case of preparedness against forthcoming epidemics, this transitional state is reflected in the observation that people accept the role of the State as a responsible authority but in parallel try to formulate their personal opinion on upcoming risks. Another outcome of this study is the realization that the public distinguishes trust related to belief in the objectivity of authorities (“good will” and lack of bias) and trust related to confidence in the competence of authorities [14]. A large number of respondents believed that the H1N1 vaccination campaign was grounded in honesty and good will, yet the actual behavior during the epidemic suggests that the public did not accept the judgment of the expert authorities.

The emergence of a vaccination-reflexive population, which is also manifested in the changing attitudes of the public to MMR vaccination, HPV vaccination, and childhood vaccination protocols [20,24], deserves careful consideration. Reasoned-assessment and reflexivity towards vaccination are by no means identical to making the right decisions. In the first paper in this series [7] we have clearly stated that in many cases (about 30%) reasoned non-compliance with swine-flu vaccination was based on wrong assumptions (for example, “I am eating a healthy diet and therefore I am protected against flu”).

The trends identified in this study may, therefore, have dangerous implications for the cause of public health. Reflexive assessment of vaccination programs by lay individuals rely, in most cases, on personal perceptions of risks and is most likely based on self-interests. Lay individuals are less likely to take into consideration group-interests related to vaccines, and are less receptive to concepts such as vaccination for the sake of the more vulnerable or vaccination aimed at achieving herd immunity. In addition, assessment of vaccination programs and most specifically new vaccination programs requires high degrees of knowledge and understanding that are in many cases beyond the capability of even the most sophisticated lay individuals. In addition, a judgmental public is more likely to reject a “new vaccine”. This is precisely the scenario where the stakes are highest – an emerging infection for which there is no effective treatment other than a newly developed vaccine.

In spite of the anticipated dangers, vaccination-reflexivity can be harnessed to enhance the benefits from vaccination. “Vaccination-reflexivity” should be distinguished from “Anti-vaccination”. Recent studies suggest that only 2-8% of the population in developed countries can be defined as “hard anti-vaccinators”, whereas the number of individuals that exhibit reflexive attitudes is on the rise [20,24]. Anti-vaccination movements, which have accompanied vaccination from its very beginning, are mostly based on ideologies, beliefs, and emotional approaches that magnify dangers, and therefore are less amenable to change. Reflexive-vaccination, on the other hand, is based on assessment and evaluation and could be targeted by appropriate communication programs.

Our findings, which indicate that the reflexive public did not lose its trust in the genuine motivation of authorities, and did appreciate the difficulty faced by authorities in dealing with high uncertainties, suggest that the public could be receptive to risk-communication messages. Nevertheless, these messages should take into consideration the fact that the public does not accept recommendations in a non-conditional way.

## Conclusions

Policy makers should take into account the fact that the public does not accept authority’s recommendations in an unconditional manner. Modern individuals tend to evaluate such recommendations and formulate their mode of response accordingly. Nevertheless, in certain cases the public may respond in a non-reflective way and may delegate this responsibly to authorities in an uncontested manner. The shift between the two reactions is probably not predictable and will depend on the specific characteristics of the risks involved. This leaves the policy makers with the complex challenge of attending to the needs of a passive non-involved public as well as the needs of an opinionated, reflexive public.

The emergence of a “trusting-yet-reflexive” public calls for the redefinition of the vaccination dialogue between the authorities and the public. This dialogue should be respectful towards lay-judgment and assume that individuals are well motivated. Special attention should be given to bridging the gap between expert-knowledge and lay-knowledge. Lay individuals should not be viewed as scientific illiterates but as partially-informed agents, to which new information should be communicated. This becomes a special challenge when an emerging epidemic is identified. In this case, expert authorities should communicate to the public the inherent uncertainties related to the extent of the danger and to the effectiveness of countermeasures, and full transparency should be maintained.

## Competing interests

The authors declare that they have no competing interests.

## Authors’ contributions

The study was designed by GK, LL, and BV. Data analysis was conducted by VB, BV, GK, LL, and GS. VB performed the statistical analyses and designed the tables. The manuscript was written by BV with the help of all authors. All the authors have read and approved the final manuscript.

## Authors’ informations

Liat Lerner-Geva MD, PhD, is a board-certified physician in Epidemiology and Public Health with special emphasis on reproductive epidemiology. Since 2001, she has been director of the Women and Children’s Health Research Unit at the Gertner Institute for Epidemiology and Health Policy Research (Ltd,) and since 2009 an associate professor at the School of Public Health, Sackler Faculty of Medicine, Tel-Aviv University.

Gilead Shenhar retired as Colonel from Israel Defense Force (IDF) serving in active duty from 1976 to 2003. In his last position he was the head of doctrine and development in civil protection. Today he is a senior researcher at Gertner Institute, and academic coordinator and teacher at Tel-Aviv University in the Emergency and Disaster Management program. Shenhar also serves as a senior spokesman for Israeli civil protection during emergencies. He has been a member of several delegations to disaster areas with the IDF and UNDAC.

Valentina Boyko, MSc, is currently the senior biostatistician at the Women and Children’s Research Unit, at the Gertner Institute for Epidemiology and Health Research, Sheba Medical Center, Israel. She has extensive experience in all fields of biostatistics, with particular interest in women’s health, perinatology, and cardiovascular diseases.

Giora Kaplan was born in Argentina and has lived in Israel since 1966. He studied Sociology, Management of Human Services, and has a Ph.D. in Public Health. Since 1975 he has been a researcher in the health system, and has been a senior researcher at the Gertner Institute for Epidemiology and Health Policy Research since its foundation. Dr. Kaplan currently heads Gertner’s Psychosocial Aspects of Health Division. His primary research interests include: coping with illness, cultural aspects of health, social consequences of health policy, and consultation with the public regarding issues of health ethics and policy.

Baruch Velan is a career scientist involved in a variety of projects related to the mechanisms of microbial pathogenesis, interactions between hosts and invading microorganisms, immune-evasion strategies, as well as development of classical and recombinant vaccines. Dr. Velan has conducted most of his scientific activities at the Israel Institute for Biological Research, where he also assumed several executive functions. At present, Dr. Velan is studying various aspects in vaccination ethics at the Gertner Institute in the Sheba Medical Center.
